# Low-frequency pitch sensitivity and speech perception performance in adult cochlear implant users fitted with fine structure strategies

**DOI:** 10.1007/s00405-025-09449-y

**Published:** 2025-05-20

**Authors:** Patrizia Mancini, Yılmaz Odabaşı, Ginevra Portanova, Francesca Yoshie Russo, Giannicola Iannella, Hilal Dincer D’Alessandro

**Affiliations:** 1 Department of Sense Organs, Sapienza University of Rome, Rome, Italy; 2 Department of Audiology, Faculty of Health Sciences, Hacettepe University, Ankara, Turkey; 3 Department of Audiology, Faculty of Health Sciences, Istanbul University-Cerrahpaşa, Istanbul, Turkey

**Keywords:** Cochlear implant, Temporal fine structure, Temporal pitch, place pitch, Speech perception

## Abstract

**Purpose:**

Cochlear implant (CI) users may perceive pitch changes via repetition rate (RP) and place-of-stimulation (PP) coding mechanisms. This study investigated whether CI users fitted with fine structure (FS) strategies can discriminate low-frequency (LF) pitch changes via RP and whether RP performers show better speech recognition than PP performers.

**Methods:**

Thirty postlingually deafened adult CI users (15 unilateral, 15 bilateral) participated in this study. LF pitch discrimination linked to temporal fine structure (TFS) sensitivity was assessed with the Disharmonic Intonation test (A§E psychoacoustic test suite), while speech perception was evaluated with phonetically balanced words and everyday sentences (both in quiet and noise), and the Italian Matrix test (in adaptive mode).

**Results:**

Just noticeable differences (JNDs) in RP performers were significantly better compared to PP JNDs (*p* < 0.001, *r* = 0.80). No significant differences were found between unilateral and bilateral CI users for LF pitch perception (*p* > 0.05). Within-group comparisons (better ear versus bilateral listening) revealed that better ear JNDs were not significantly different from the bilateral performance (*p* > 0.05), whereas significant bilateral benefit was observed for word recognition score (WRS) + 10 (*p* = 0.016, *r* = 1.00), WRS + 5 (*p* = 0.021, *r* = 1.00), and Matrix performance (*p* = 0.033, *r* = 0.80). Speech recognition in noise was significantly better in RP performers compared to PP performers across multiple tests, including WRS + 10 (*p* = 0.002, *r* = 0.90), WRS + 5 (*p* = 0.001, *r* = 0.90), and the Matrix test (*p* = 0.03, *r* = 0.60).

**Conclusion:**

These findings highlight the advantages of FS coding strategies in improving TFS sensitivity and speech perception in complex listening environments.

**Supplementary Information:**

The online version contains supplementary material available at 10.1007/s00405-025-09449-y.

## Introduction

Cochlear implants (CIs) became an effective treatment for postlingually deafened adults in terms of restoring functional hearing by electrically evoking the auditory system, bypassing deficient transducer structures and inducing action potentials at the auditory nerve sites [1]. Outcomes from the majority of adult CI recipients showing aided hearing thresholds within/above the speech banana as well as speech recognition scores above 80% for open-set sentences presented in quiet can be cited as evidence for this fact. However, both clinical outcomes and patients’ self-reports still reflect difficulties for speech perception in noisy listening conditions that are typical during everyday life [2, 3].

Conventional CI systems incorporating multiple-electrode assemblies are designed to mimic tonotopic principle of the healthy cochlea. Such an approach helps to sufficiently convey envelope cues, i.e., slow rate amplitude fluctuations in speech signals over time, whereas Temporal Fine Structure (TFS) cues that are characterized by rapid oscillations in complex acoustic signals are mostly discarded. In fact, such a working principle in CI technology has significant negative effects on spectrotemporal coding mechanisms, resulting in significant limitations in low-frequency (LF) pitch perception linked to LF TFS sensitivity [3].

Pitch perception studies in the field of Neurophysiology suggest two coding mechanisms: phase locking (temporal or rate pitch - RP) and place coding (spectral or place pitch - PP). Both mechanisms are believed to be frequency sensitive. More precisely, high-frequency (HF) processing is mainly dominated by place coding mechanism, which is based on spectral excitation of nerve fibers. Contrariwise, LF pitch encoding is dominated by phase locking, a time-based mechanism that locks onto the TFS in complex acoustic signals [4, 5]. The significant role of the availability of LF pitch and TFS cues in speech understanding is reflected by studies in tonal languages where changes in Fundamental Frequency (F0) over time typically result in semantic differences. Recently, the perception of F0 information, related to pitch perception, has attracted a lot of interest in CI research because of its role in complex listening performance such as music listening and speech-in-noise. Indeed, TFS sensitivity is shown to be crucial for speech perception in fluctuating background sounds, in speaker identification, and music perception performances [3, 6]. Such complex listening performances demanding higher TFS sensitivity are very challenging for CI users. Nevertheless, there exists considerable interindividual performance variability whilst reasons for such outcome differences, especially in complex listening activities, continue to be a hot topic of recent CI studies.

Among many individual-specific factors such as demographic, audiological, and cognitive profile, the effects of device-specific technical characteristics on performance differences remain to be one of the most challenging research aspects in postlingually deafened adult CI users [7]. The device type, more specifically the sound coding strategy, may play a significant role in patient performance. Sound coding by strategies such as Fine Structure (FS) that is based on channel-specific real-time filters may provide better TFS cues rather than those that are based on Fast Fourier Transform analysis shown to result in temporal smearing [8]. Moreover, FS strategies have an extended LF range compared to other commercially available coding strategies. Indeed, FS strategies aim to enhance sound perception by preserving fine temporal details of acoustic signals, particularly in LF regions where temporal cues are crucial for pitch perception, speech understanding, and music appreciation [9, 10]. However, the sample compositions in previous studies did not allow studying aspects such as the effect of the spectral range, the number of stimulating electrodes, stimulation rate, and temporal envelope extraction on LF pitch/TFS outcomes in CI populations.

CI users may discriminate pitch changes with RP (repetition rate of stimulation on one channel) and PP (the site of stimulation is changed while keeping the stimulation rate constant) coding mechanisms [11]. RP refers to the ability to distinguish pitch changes within the same electrode whilst PP refers to the pitch discrimination ability resulting from a change in the coding electrode as the corresponding spectral band moves to the adjacent electrode and provides a place cue. The principal aim of the present study was to investigate whether CI users fitted with FS strategies are able to discriminate LF pitch changes by RP. It was expected that RP performers would be those showing significantly better speech-in-noise recognition skills than PP performers. Furthermore, the effects of demographic characteristics, speech test material/mode, and the listening mode are evaluated on the present outcomes.

## Materials and methods

The present study was carried out in accordance with the ethical requirements of the 1964 Declarations of Helsinki, its later amendments, the Epidemiological Good Practice Guidelines of the International Conference of Harmonization, and the existing legislation in Italy. The ethical approval was obtained from the local ethics committee of Sapienza University of Rome (protocol no 259/2020) and all patients gave informed consent for their study participance.

### Participants

The study participants were 30 postlingually deafened adult CI users (15 female and 15 male) aged between 20 and 83 years (Mean = 58yrs, SD = 16.9) at the time of testing. Participation was on a voluntary basis during their annual visit. The mean duration of CI use was 4.7 years (ranging from 6 months to 15 years, SD = 3.3). The mean duration of hearing loss was 23 years (ranging from 6 months to 64 years, SD = 15.2). Age at implantation ranged from 18 to 77 years (Mean = 53.5yrs, SD = 16.5). All participants were native Italian speakers and did not show any cochlear or auditory nerve anomalies. Demographic characteristics for the study participants are shown in Table 1.

**Table 1:** Demographic characteristics for the study group

Participants		Duration of HL (years)	Age at CI (years)	Age at Test (years)
		Mean (SD)	Mean (SD)	Mean (SD)
Unilateral CI (*n* = 15)		26.9 (15.9)	59.1(13.1)	64.7 (12.4)
Bilateral CI (*n* = 15)		22.3 (15.8)	50.8 (18.4)	54.9 (18.3)
**Device** **(** ***N*** **= 45)**		***n*** ** of subjects (%)**		
Receiver type	Combi40+	2 (4.4%)		
	Pulsar CI100	2 (4.4%)		
	Sonata	2 (4.4%)		
	Synchrony	39 (86.7%)		
Electrode type	Flex24	4 (8.9%)		
	Flex28	28 (62.2%)		
	Standard	13 (28.8%)		
Strategy	FSP	6 (13.3%)		
	FS4	31 (69%)		
	FS4-p	8 (17.7%)		

Reported variables are expressed as Mean and SD. CI = Cochlear Implant, HL = Hearing Loss, R = Right, L = Left, n = number, M = Male, F = Female, FSP = Fine Structure Processing of the most two apical channels, FS4 = Fine Structure Processing Strategy expanded to four apical channels, FS4-p = FS4 in parallel stimulation

The overall study group consisted of both unilateral (*n* = 15) and bilateral (*n* = 15) CI users. Five of the bilateral CI users were implanted sequentially whilst 10 of them received their implants simultaneously. For sequential bilateral users, the time interval for the second implant was < 4 years (Mean = 2.9yrs, SD = 1.6). There were no statistically significant differences for duration of hearing loss, age at CI, age at test, and duration of CI use between unilateral and bilateral subgroups (*p* > 0.05).

Both unilateral and bilateral CI users did not show any LF residual hearing. The pure tone averages (PTAs) were above 85 dB for octave frequencies between 125 and 1000 Hz for both ears. For unilateral CI users, the average aided Sound Field (SF) threshold for octave frequencies between 125 and 8000 Hz was 31.3 dB HL (SD = 6.7). For bilateral CI users, the corresponding values on the right and left side were 34.3 dB HL (SD = 4.4) and 35 dB HL (SD = 5.7), respectively. The mean bilateral CI threshold was 29.6 dB HL (SD = 3.5). Statistically significant differences were found between bilateral and left/right only (*p* = 0.001, *r* = 0.85) as well as between bilateral and better ear (BE) performances (*p* = 0.002, *r* = 0. 88). PTA differences between unilateral and bilateral users as well as within the bilateral subgroup for simultaneous versus sequential CI were not statistically significant.

Implant characteristics are categorized by receivers, electrode types, and processing strategies (Table 1). More detailed information for the participants’ implant characteristics including the CI side, number of active electrodes, FS coding strategy, and the channel’s number/bandwidth corresponding to F0 of 200 Hz are reported in Appendix 1. For the overall study sample (*N* = 45 ears), all but two ears had full insertion of all 12 electrodes at surgery. Partial insertion of electrodes happened in two cases (participants B4 and B15). Participant B4 had 11 electrodes and participant B15 had 10 electrodes inserted on their left sides during the surgery. The total number of active channels at the time of testing were 12 in 55.5% (*n* = 25 ears), 11 in 20% (*n* = 9 ears), 10 in 15.5% (*n* = 7 ears), 9 in 6.7% (*n* = 3 ears) and 8 in 2,2% (*n* = 1 ears). All but two deactivated electrodes were the most basal ones. Deactivation of electrodes other than the most basal ones was observed only in two cases (participants B10 and B15). Participant B10 had the 5th electrode deactivated on the right side while participant B15 had the 6th electrode deactivated on the left side along with the 12th electrode. The numbers of FS channels were 4 in 82.2% (*n* = 37 ears), 3 in 4.4% (*n* = 2 ears), and 2 in 13.3% (*n* = 6 ears). F0 of 200 Hz was corresponding to the 1st most apical channel in 42.2% (*n* = 19 ears) and to the 2nd most apical channel in 57.8% (*n* = 26 ears) of the cases. The lower frequency limits were 100 Hz for 84.4% (*n* = 38 ears), 70 Hz for 8.8% (*n* = 4 ears), 200 Hz for 4.4% (*n* = 2 ears), and 150 Hz for 2.2% (*n* = 1 ear).

### Test procedures

During testing, all participants were requested to use their daily CI settings. All assessments were conducted in a sound-treated room. The participants sat on a chair, in front of a loudspeaker (Indianaline, Coral electronic, Italy) 1 m away from them at 0° azimuth. Testing was performed for a total of 45 ears from both unilateral and bilateral users plus for the bilateral listening condition in the latter case. The bilateral CI users were tested bilaterally first and then randomly on the single sides. Assessments lasted approximately 1 h, and participants could request to take a break whenever needed.

For hearing threshold assessment, standard audiological testing was performed at octave frequencies from 125 to 8000 Hz. A warble tone from Aurical audiometer (Otometrics Taastrup, Denmark) with TDH39 professional headphones was used for unaided thresholds. CI thresholds were also measured for the same octave frequencies, with the same audiometer using the loudspeakers as the above-mentioned protocol.

### Pitch perception assessment

For evaluating LF pitch perception, Disharmonic Intonation (DI) test from the A§E psychoacoustic test suite is used. This test aims to determine the Just Noticeable Difference (JND) for LF pitch changes, thought to be linked to availability of TFS cues. The task for the participants is to discriminate between two consecutive stimuli where one non-intonating sound is contrasted to an intonating sound. The non-intonating sound is a harmonic complex signal of an F0 at 200 Hz and its three higher harmonics at lower intensities (-6 dB at 2F0, -12 dB at 3F0, and − 18 dB at 4F0). The intonating sound is characterized by a frequency sweep of F0 [F0 to F0+∆F] whilst the higher harmonics do not sweep and remain stable. This results in disharmonic intonation. The sweep begins at 330ms after the start of the signal and lasts for 120ms. Each stimulus lasts 600ms and the two consecutive stimuli are separated with a 500ms inter-stimulus interval. White noise is added to the stimuli (SNR + 10.9 dB) resulting in the stimuli to sound more natural whilst intensity roving (± 2 dB) was applied to control for the use of loudness cues by the participants. The stimuli were administered at 70 dB SPL and the participants were asked to respond whether the two consecutive sounds were the same or different. Testing was always preceded by training in order to familiarize the participants with the task. ∆F ranged between 0 and 214 Hz, starting from 41 Hz and changing adaptively until estimating the 50%-point on a participant’s psychometric curve. ∆F at 0 Hz means no change between two stimuli and serves as an internal check to increase the reliability of the test. Whenever a JND could not be achieved within 100 trials, it was set to 220 Hz, above the maximum ΔF at 214 Hz [5, 12].

### Speech perception assessment

For evaluating the participants’ speech perception performances both in quiet and in noise with a fixed SNR, standard phonetically balanced bisyllabic words and everyday sentences developed for Italian adult listeners were used [13]. Word and sentence recognition tests in quiet/noise were performed with the speech signal fixed at 65 dB SPL, including both + 10 and + 5 dB SNR presentations.

For the adaptive speech in noise testing, the Italian Matrix test is used in an open-set response format. The test consists of semantically unpredictable but syntactically fixed sentences (name-verb-numeral-noun-adjective, e.g., ‘*Simone manda venti porte rosse’*, which is Italian for ‘Simone sends twenty red doors’). Masking noise with the same long-term spectrum of the speech material is generated through 30-fold overlapping of all the sentences. For the present assessments, each test list consisted of 30 sentences with the noise level fixed at 65 dB SPL. An SNR of 0 dB was administered initially and the subsequent speech level changed adaptively until estimating the SNR where 50% of words were repeated correctly. Testing was preceded by two practice lists to minimize learning effects [14]. A valid result was defined as a signal to noise ratio of up to 20 dB, the SNR value considered easy speech perception with still perceivable noise [15, 16].

### Statistical analysis

Data analysis was carried out with the Statistical Package for Social Sciences (SPSS version 25.0, IBM Corporations, Chicago, IL, USA). The Shapiro-Wilk test showed that the DI and Matrix data were not normally distributed (*p* ≤ 0.001); hence, non-parametric statistical tests were adopted. Descriptive statistics were reported as median and minimum/maximum values.

For each participant, the electrode location corresponding to F0 of 200 Hz coding (usually the first or second electrode) is determined. Based on the individual frequency distribution through the active electrodes and DI outcomes, RP and PP performers were defined as participants/ears being able to discriminate pitch changes either within the same electrode or by a sweep to an adjacent one, respectively. Percentage of performers showing within normal range JNDs were calculated (defined as DI scores ≤ 10 Hz by a previous study by Vaerenberg et al. [5].

For speech perception in quiet/noise (fixed SNR), the percent values of correct responses were transformed to Rationalised Arcsine Units (RAUs) with the aim to avoid the ceiling effects [17]. For the bilateral CI participants, the BE was determined by a better DI score, except for a patient with similar JNDs at 220 Hz for both ears. In this case, the BE was decided according to the better Matrix performance.

The Mann-Whitney U test was carried out for unilateral versus bilateral as well as for RP versus PP performance comparisons (for both pitch and speech perception). Wilcoxon test was performed for BE versus bilateral comparisons along with the effect size to define the magnitude of the relationship between variables. The effect size was calculated using the Rosenthal formula r = Z/$\:\sqrt{N}$ (very low = 0.00 to 0.20, low = 0.20 to 0.40, moderate = 0.40 to 0.60, strong = 0.60 to 0.80 and very strong = 0.80 to 1.00) [18].

Spearman bivariate correlations were performed to analyze the relationship between DI outcomes, demographic (age, age at implant, duration of deafness and duration of CI experience) and audiological (SF and speech perception) variables. The cutoff level for statistical significance was set to 0.05.

## Results

### Pitch perception

**Fig. 1:**
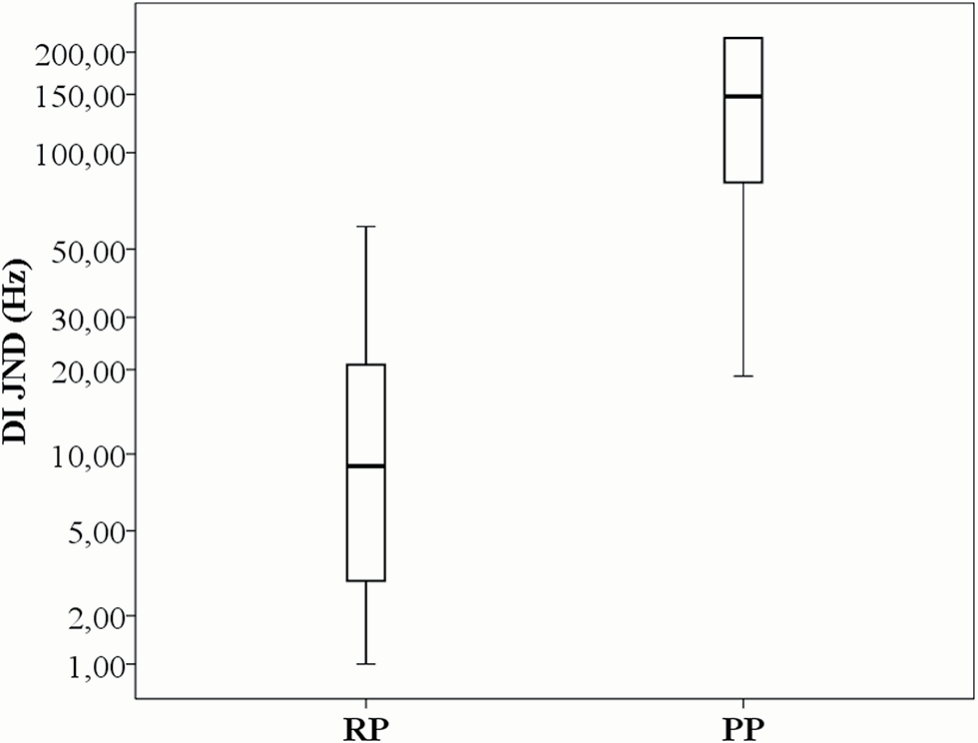
DI JNDs from the overall study group (*N* = 45 ears) classified as RP (*n* = 27 ears) and PP (*n* = 18 ears) performers. Median values for RP and PP are 9 Hz and 148 Hz, respectively

**Fig. 2:**
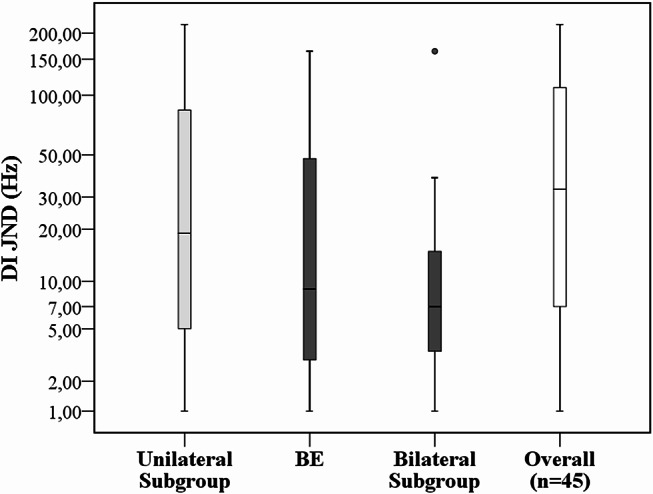
DI JNDs based on listening modes. Unilateral (*n* = 15), bilateral (*n* = 15), better ear (*n* = 15) and overall (*N* = 45) median values are 19, 7, 9 and 33 Hz, respectively

Table 2 represents the median DI JNDs including minimum/maximum scores and RP/PP perception ability for the overall study group (*N* = 45 ears) as well as for the unilateral versus bilateral subgroups (see also Figs. 1 and 2).

**Table 2:** Descriptive statistics concerning median DI scores and FS coding channels

Group	DI JND (Hz) Median (min-max)	RP (min-max) [*n*]	PP (min-max) [*n*]	FS coding channels [*n*]
Overall (*N* = 45 ears)	33 (1–220)	9 (1–57) [27]	148 (19–220) [18]	4 [37] 3 [2] 2 [6]
Unilateral CI (*n* = 15)	19 (1–220)	11 (1–57) [10]	220 (19–220) [5]	4 [10] 2 [5]
Bilateral CI (*n* = 15)	7 (1–220)	9 (1– 57) [17]	109 (64–220) [13]	4 [27] 3 [2] 2 [1]
BE	9 (1–164)	7 (1– 57) [10]	122 (64–164) [5]	4[15]

Values are median (min–max) scores for pitch discrimination of the whole dataset. Rate Pitch refers to the ability to discriminate pitch changes in the same apical electrode (1st or 2nd), whilst Place Pitch refers to the ability to discriminate pitch due to shift into the next adjacent electrode. DI = Disharmonic Intonation, JND = Just Noticeable Difference, RP = Rate Pitch, PP = Place Pitch, FS = Fine Structure, BE = Better Ear determined by scores at DI or Matrix test in bilateral CI users

The median JND from a total of 45 ears was 33 Hz (min = 1, max = 220 Hz). RP and PP JNDs were 9 Hz (60%, *n* = 27 ears) and 148 Hz (40%, *n* = 18 ears), respectively (see Appendix 1 for the individual implant characteristics with the RP/PP ability). As shown in Fig. 4.1, RP JNDs were significantly lower (better) than PP JNDs, showing a very strong effect size (*p* < 0.001, *r* = 0.80). For the overall group, DI scores from 15 ears (33.3%) were within the clinical normal zone (≤ 10 Hz), all from RP performers. This score corresponded to 55% of RP performance.

DI JNDs for unilateral versus bilateral CI subgroups did not show any statistically significant performance differences (*p* > 0.05). For the unilateral subgroup, RP versus PP performers were 66.6% and 33.3%, respectively. These performances in the bilateral subgroup were 80% (RP on both sides or RP on one side and PP on the other side) and 20% (PP on both sides), respectively. For the BE alone, RP versus PP performances were 73.3% and 26.7%, respectively. BE JNDs were not significantly different from the bilateral performance (*p* > 0.05).

In the unilateral subgroup, performers within the clinical normal zone were 33.3%. The corresponding values in the bilateral subgroup and BEs were 33.3% and 53.3%, respectively. Demographic and audiological data did not show any statistically significant effects on overall DI results (*p* > 0.05).

### Speech perception

Table 3 represents the median (min–max) speech perception scores for the overall group (*N* = 45 ears) as well as for the unilateral versus bilateral subgroups.

**Table 3:** Speech perception scores for the overall group (*N* = 45 ears) as well as for the unilateral versus bilateral subgroups

Group	WRS_q % (min-max)	WRS + 10% (min-max)	WRS + 5% (min-max)	SRS_q % (min-max)	SRS + 10% (min-max)	SRS + 5% (min-max)	Matrix (dB SNR) (min-max)
Overall (*N* = 45)	76 (28–100)	42 (10–88)	16 (0–80)	84 (40–100)	46 (0–100)	17 (0–90)	7.0 (-4.2–20)
Unilateral CI (*n* = 15)	80 (52–95)	41 (2–88)	12.5 (0–50)	90 (60–100)	45 (10–100)	15 (0–70)	8.2 (-1.9–20)
Bilateral CI (*n* = 15)	90 (65–100)	56 (38–88)	36.5 (20–80)	90 (80–100)	70 (30–100)	40 (0–100)	1.3 (-4.2–7.2)
BE	79 (65–92)	44 (10–70)	20 (0–60)	85 (60–100)	50 (10–90)	15 (0–90)	4.1 (-3–9.5)

Median (min-max) scores are reported. WRS_q = Words Recognition Score in Quiet, WRS + 10 = Words Recognition Score at 10 dB SNR, WRS + 5 = Words Recognition Score at 5 dB SNR, SRS_q = Sentence Recognition Score in Quiet, SRS + 10 = Sentence Recognition Score at 10 dB SNR, SRS + 5 = Sentence Recognition Score at 5 dB SNR, CI = Cochlear Implant, RP = Rate Pitch, PP = Place Pitch, BE = Better Ear determined by scores at DI or Matrix test in bilateral CI users

For the overall group, the median speech perception scores were 76% for Word Recognition Score in Quiet (WRS_q), 42% for Word Recognition Score at 10 dB SNR (WRS + 10), and 16% for Word Recognition Score at 5 dB SNR (WRS + 5). The corresponding values for sentence recognition were 84% for Sentence Recognition Score in Quiet (SRS_q), 46% for Sentence Recognition Score at 10 dB SNR (SRS + 10), and 17% for Sentence Recognition Score at 5 dB SNR (SRS + 5). The median SRT for the Matrix test was 7.0 dB SNR.

For the unilateral subgroup, the median speech perception scores for WRS_q, WRS + 10 and WRS + 5 were 80%, 41% and 12.5%, respectively. The corresponding values for sentences were 90%, 45% and 15%, respectively. The median SRT from Matrix test was 8.2 dB SNR. For the bilateral subgroup, these scores were 90%, 56% and 36.5% for words versus 90%, 70% and 40% for sentences, and 1.3 dB SNR for the Matrix test. BE scores were 79%, 44% and 20% for words versus 85%, 50% and 15% for sentences, with a median Matrix SRT at 4.1 dB SNR.

Performance comparisons between unilateral and bilateral subgroups showed statistically significant differences for WRS + 10 (*p* = 0.047, *r* = 0.80), WRS + 5 (*p* = 0.024, *r* = 0.90), SRS + 5 (*p* = 0.029, *r* = 0.90) and Matrix tests (*p* = 0.002, *r* = 1.00). Within-group comparisons for the bilateral subgroup (BE versus bilateral listening) revealed statistically significant differences for WRS + 10 (*p* = 0.016, *r* = 1.00), WRS + 5 (*p* = 0.021, *r* = 1.00) and Matrix test (*p* = 0.033, *r* = 0.80). Demographic and audiological data from the present sample did not show any statistically significant effects on speech perception scores (*p* > 0.05).

### Effects of rate/place pitch on speech perception

**Fig. 3:**
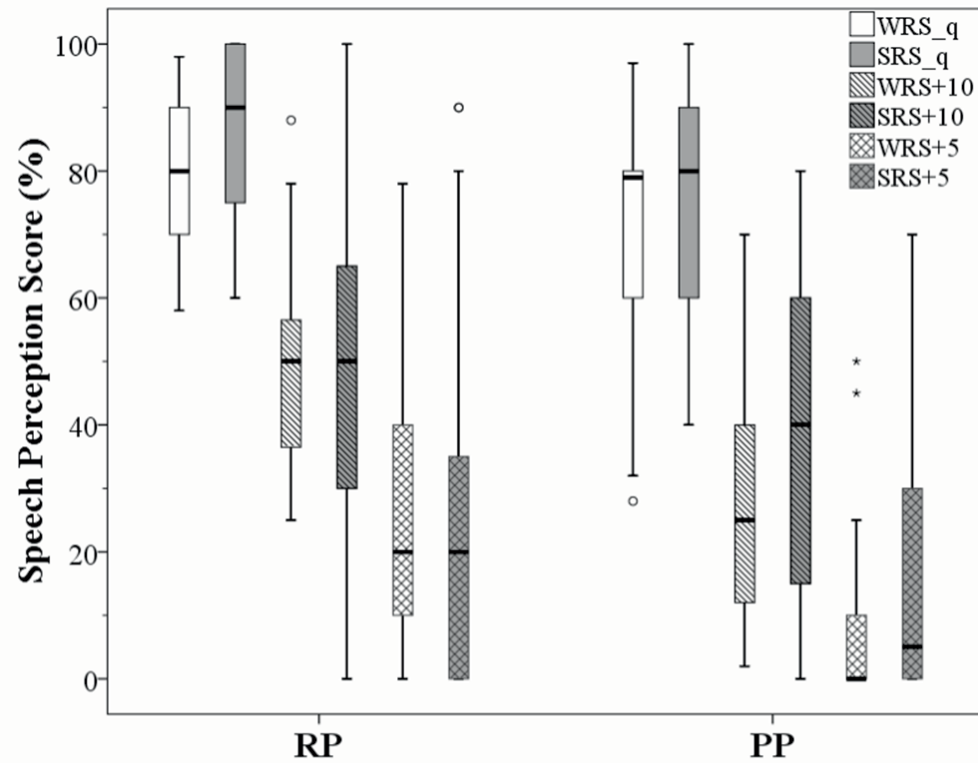
Speech perception scores in quiet/noise for both words and sentences, classified as RP and PP performers. RP = Rate Pitch, PP = Place Pitch, WRS_q = Words Recognition Score in Quiet, SRS_q = Sentence Recognition Score in Quiet, WRS + 10 = Words Recognition Score at 10 dB SNR, SRS + 10 = Sentence Recognition Score at 10 dB SNR, WRS + 5 = Words Recognition Score at 5 dB SNR, SRS + 5 = Sentence Recognition Score at 5 dB SNR

**Fig. 4:**
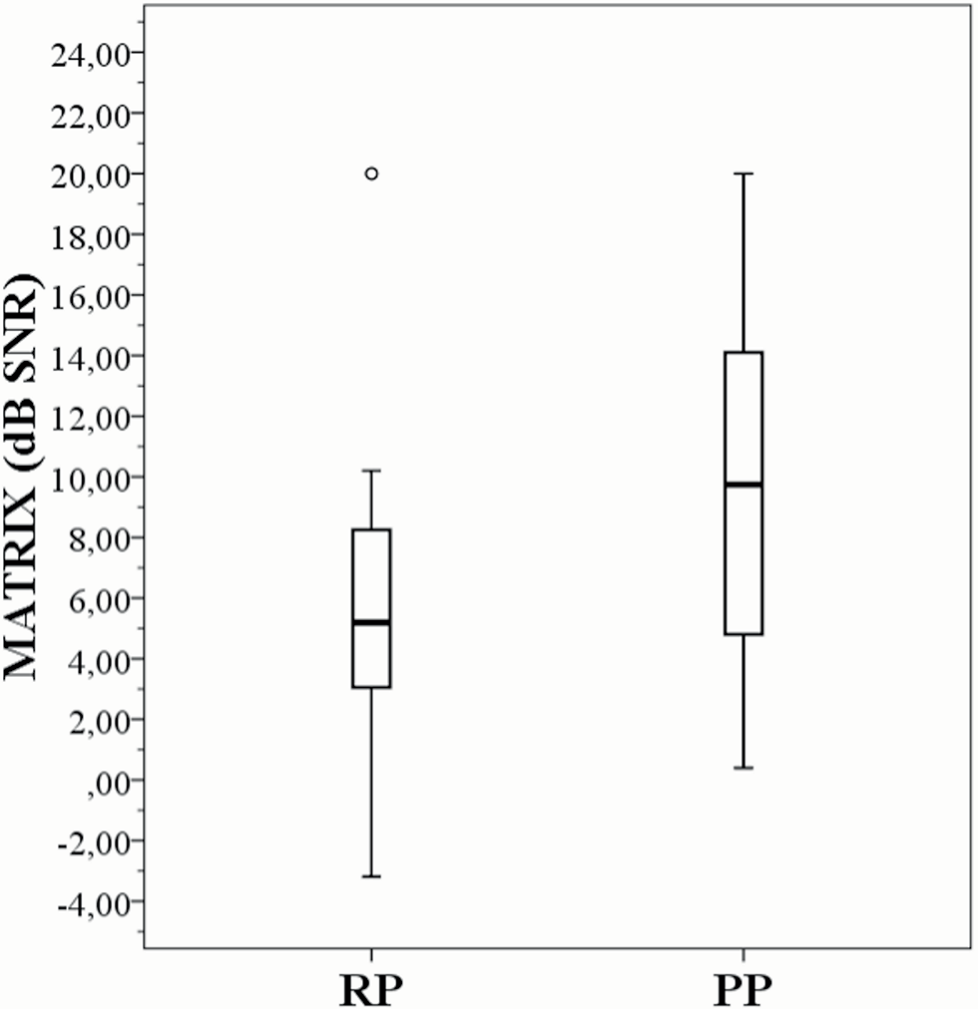
Matrix scores classified as RP and PP performers. Median SRTs are 5.2 and 10 dB SNR, respectively. RP = Rate Pitch, PP = Place Pitch

Speech perception scores from the overall group (*N* = 45 ears) were divided into two subgroups based on their pitch perception ability (RP versus PP performers) (see Table 4). Figure 3 represents speech perception scores in quiet/noise for both words and sentences whilst Fig. 4 illustrates Matrix results, classified as RP and PP performers.

**Table 4:** Speech perception scores classified as RP and PP performance

	RP (*n* = 27 ears)	PP (*n* = 18 ears)	Rosenthal’s *r* (*p*)
WRS_q (%)	80 (58–98)	80 (28–97)	0.40 (0.100)
WRS + 10 (%)	50 (25–88)	30 (2–70)	**0.90 (0.002)**
WRS + 5 (%)	20 (0–78)	0 (0–50)	**0.90 (0.001)**
SRS_q (%)	90 (60–100)	80 (40–100)	0.60 (0.060)
SRS + 10 (%)	50 (0–100)	40 (0–80)	0.50 (0.200)
SRS + 5 (%)	20 (0–90)	10 (0–70)	0.20 (0.700)
Matrix (dB SNR)	5.2 (-3.2–20)	10 (0.4–20)	**0.60 (0.030)**

Median (min–max) scores for speech perception. Bold values show statistically significant differences at *p* < 0.05. RP = Rate Pitch, PP = Place Pitch, WRS_q = Words Recognition Score in Quiet, WRS + 10 = Words Recognition Score at 10 dB SNR, WRS + 5 = Words Recognition Score at 5 dB SNR, SRS_q = Sentence Recognition Score in Quiet, SRS + 10 = Sentence Recognition Score at 10 dB SNR, SRS + 5 = Sentence Recognition Score at 5 dB SNR

Group comparisons for RP/PP performers showed statistically significant differences for WRS + 10 (*p* = 0.002, *r* = 0.90), WRS + 5 (*p* = 0.001, *r* = 0.90) and Matrix tests (*p* = 0.03, *r* = 0.60) (see Table 3). Demographic and audiological data did not significantly differ for speech perception scores from RP and PP performers. The demographics did not show any statistically significant effects on RP and PP performances.

## Discussion

Complex listening performance has received significant attention in the field of cochlear implantation, particularly within the last fifteen years. The focus has shifted from simple speech recognition in quiet based on isolated words to more complex aspects of listening, such as how CI users deal with the unpredictability of speech and experience real-world listening environments where noise is inevitable. Such studies have been motivated by significant advances in CI technology and the development of ecologically more valid assessment tools mimicking everyday listening situations. Researchers have been exploring how CI users process and understand spoken language in real-life listening situations, especially when faced with challenging listening tasks such as speech with varying rate, background noise, or unfamiliar accents [19–23]. Despite a dramatic performance deterioration for complex listening tasks in the majority of CI users, outcomes reflect remarkable interindividual variability, whilst reasons for such performance differences largely remain unknown for aspects regarding device characteristics. Indeed, it is reasonable to expect a significant effect of device type on outcome differences, e.g., sound coding strategy may play a significant role in patient performance, however, such research aspects continue to be very challenging due to presence of several individual-specific confounding factors such as a patient’s demographic, audiological, and cognitive profile [7].

Recent research in CI technology focuses on attempts to overcome technical barriers and optimize patient performance. The working principle of conventional CI technology is known to be based on mimicking tonotopic organization of healthy cochlea. However, this approach based on conveying envelope information of complex acoustic signals results in limitations for transmission of TFS cues, which are known to be dominant in the transmission of acoustic cues below 1000 Hz and thereby, being significantly linked to LF pitch perception. TFS sensitivity is shown to be specifically crucial for complex listening performance such as speech-in-noise and music perception. Indeed, demand for higher TFS sensitivity is one of the most important aspects leading to performance difficulties in such complex listening situations for CI users [3].

A promising approach to improve LF pitch and TFS sensitivity in CI users has been realized with the development of FS strategies that are based on extending phase-locked stimulation and using channel-specific pulse rate. A zero-crossing detector is employed, and a maximum of four apical channels convey rate information. In the domain of LF channels where phase-locking is naturally effective, FS strategies modulate the timing of current pulses according to the instantaneous phase of the incoming acoustic signal instead of using fixed, biphasic current pulses, which are typical of Continuous Interleaved Sampling (i.e., CIS). For mid-to-high frequencies, FS strategies rely on place coding like other CI strategies [10, 24]. This dynamic approach is believed to improve TFS representation, making rate pitch cues more accurate, thereby improving speech-in-noise performance, music appreciation, and overall sound quality compared to traditional envelope-based strategies [9].

It is a matter of fact that novel pitch perception measures have been introduced in CI clinical research with the aim to accomplish more fine-grained analysis of coding of different acoustic components, as well as to investigate the effects of sound coding strategy and/or electrode design on speech perception, possibly assisting CI mapping [25]. One measure of pitch perception in the LF range, the DI test, has been developed to gain insight into sound coding capacities by unaided or aided listening conditions, and outcomes are expected to be indicative for phase-locking capacity and TFS sensitivity of the auditory system [5, 12].

Previous studies using the DI test have shown abnormal LF pitch perception in the majority of CI users of various sound coding strategies including those from all three device models. Surprisingly, there were some CI users obtaining outcomes nearby or within clinical normal zone (≤ 10 Hz), however, previous sample characteristics did not allow researchers to investigate any possible effects of device characteristics. Present findings obtained in a sample of FS users (median JND of 33 Hz) were promising as they were considerably better than those reported in CI alone listeners of mixed samples from previous studies (median JNDs of 139 and 147 Hz). Likewise, the percentage of normal performers was considerably higher than the previous studies (33.3% here versus 9% and 8% in the previous studies) [6, 26]. Not surprisingly, such normal zone outcomes observed in the present sample were all from RP performers (55% of present RP performers), showing the ability to discriminate pitch changes within the same electrode bandwidth, owing to changes in the frequency of stimulation. Moreover, the number of RP performers was considerably higher than PP performers. These findings may support the significant positive effects of FS coding on LF pitch sensitivity. Moreover, RP performers’ better ability of LF pitch perception is obvious, as reflected by significant differences with a very strong effect size for RP/PP group comparisons (median JNDs of 9 Hz vs. 148 Hz for RP and PP performers, respectively). Indeed, RP performers’ median JND seems to be within the clinical normal zone for the DI test, while the PP performers’ score is similar to abnormal LF pitch results observed in previous studies conducted in mixed device groups of CI alone listeners, reflecting pitch discrimination ability from a larger change, where the frequency sweep implies a spectral-band change to the adjacent electrode, thereby providing a place cue.

As expected, RP performers are those showing significantly better speech-in-noise performance than PP performers. In fact, significant differences are present for more challenging listening conditions, i.e., Matrix and words in noise performances [13, 14]. More precisely, for CI users, open-set recognition of isolated words in noise and the semantically unpredictable nature of the Matrix test might be more challenging than understanding everyday sentences, where better semantic predictability of the speech material itself may provide a significant perceptual benefit, despite the challenges of listening in noise [27, 28]. Nevertheless, overall results may support the advantages of better TFS use provided by FS coding in more challenging speech perception tasks. Indeed, the positive effects of better LF pitch and TFS coding on speech understanding in noise were expected thanks to insights provided by previous studies, indicating significantly better LF pitch and TFS sensitivity in relation to better speech understanding in noise, improved by hearing aid use in addition to CI listening [29–31]. Similar trends seem to be observed with the use of FS coding strategies, better representing fine temporal details of incoming acoustic signals.

The present sample composition allowed us to study the effects of bilateral CI listening on both LF pitch and speech perception. Both between groups (unilateral versus bilateral subgroups) and better ear versus bilateral listening comparisons reflected a binaural advantage for speech perception in noise, again for more challenging Matrix [14] and words in noise [13] performances, while differences for pitch perception did not reach statistical significance. Such results have confirmed the significant positive effects of bilateral CI use for speech perception in noise. Indeed, such significant performance differences for tasks better representing everyday challenging listening may reflect pivotal improvement in everyday auditory perception [20–22, 27] and the quality of life in CI users [32, 33].

Demographic characteristics such as duration of deafness, pre-operative hearing thresholds and amplification are well-known factors that may affect postoperative auditory performance [7]. However, these factors did not show any significant impact on the performances tested within the scope of this study. On the one hand, FS use may be the determining factor of performance in a group of postlingually deafened adult CI users, but on the other hand, such results might be due to the sample characteristics. Indeed, the main limitations of the study were the small sample size and heterogeneity of the participants. Future research in larger and more homogeneous populations would be useful to better understand the role of FS coding strategies’ effects on pitch and speech perception. Moreover, it would be interesting to measure the degree of electrode insertion in the cochlea to verify if better DI values are correlated to deeper insertions.

## Electronic supplementary material

Below is the link to the electronic supplementary material.

Supplementary Material 1

## Data Availability

The datasets generated during and/or analyzed during the current study are available from the corresponding author on reasonable request.
