# Supplementary material for: Low-frequency pitch sensitivity and speech perception performance in adult cochlear implant users fitted with fine structure strategies
Source: Eur Arch Otorhinolaryngol. 2025 May 20;282(10):5137–46. doi: 10.1007/s00405-025-09449-y (PMC12518401; doi:10.1007/s00405-025-09449-y)
Supplement: Supplementary file 1 — Supplementary Material 1 [file 405_2025_9449_MOESM1_ESM.docx]

**APPENDIX 1.** The participants’ implant characteristics in relation to their RP/PP ability.

| **ID** | **CI Ear** | **FS Coding Strategy** | **Channel’s Number / Bandwidth for F0** | **n of active channels** | **RP/PP ability** | **ID** | **CI Ear** | **FS Coding Strategy** | **Channel’s Number / Bandwidth for F0** | **n of active channels** | **RP/PP ability** |
| --- | --- | --- | --- | --- | --- | --- | --- | --- | --- | --- | --- |
| U1 | L | FSP | 1^st^ / 150 to 281 Hz | 9 | PP | B1 | R | FS4 | 2^nd^ / 198 to 325 Hz | 12 | PP |
|  |  |  |  |  |  |  | L | FS4 | 2^nd^ / 198 to 325 Hz | 12 | PP |
| U2 | L | FS4 | 1^st^ / 100 to 208 Hz | 11 | RP | B2 | R | FS4 | 2^nd^ / 198 to 325 Hz | 12 | RP |
|  |  |  |  |  |  |  | L | FS4 | 2^nd^ / 198 to 325 Hz | 12 | RP |
| U3 | L | FS4 | 2^nd^ / 198 to 325 Hz | 12 | RP | B3 | R | FS4 | 1^st^ / 100 to 208 Hz | 11 | RP |
|  |  |  |  |  |  |  | L | FS4 | 1^st^ / 100 to 237 Hz | 9 | PP |
| U4 | R | FSP | 1^st^ / 100 to 221 Hz | 10 | PP | B4 | R | FS4 | 1^st^ / 100 to 221 Hz | 10 | RP |
|  |  |  |  |  |  |  | L | FSP | 1^st^ / 100 to 250 Hz | 8 | PP |
| U5 | L | FS4 | 1^st^ / 100 to 208 Hz | 11 | RP | B5 | R | FS4 | 2^nd^ / 198 to 325 Hz | 12 | RP |
|  |  |  |  |  |  |  | L | FS4 | 2^nd^ / 198 to 325 Hz | 12 | RP |
| U6 | L | FS4 | 2^nd^ / 198 to 325 Hz | 12 | RP | B6 | R | FS4-p | 1^st^ / 100 to 208 Hz | 11 | PP |
|  |  |  |  |  |  |  | L | FS4-p | 1^st^ / 100 to 237 Hz | 9 | PP |
| U7 | R | FS4 | 2^nd^ / 198 to 325 Hz | 12 | RP | B7 | R | FS4 | 2^nd^ / 198 to 325 Hz | 12 | RP |
|  |  |  |  |  |  |  | L | FS4 | 2^nd^ / 198 to 325 Hz | 12 | RP |
| U8 | R | FS4 | 1^st^ / 100 to 221 Hz | 10 | RP | B8 | R | FS4 | 1^st^ / 100 to 208 Hz | 11 | PP |
|  |  |  |  |  |  |  | L | FS4-p | 2^nd^ / 170 to 300 Hz | 12 | PP |
| U9 | R | FSP | 2^nd^ / 198 to 325 Hz | 12 | RP | B9 | R | FS4-p | 2^nd^ / 198 to 325 Hz | 12 | RP |
|  |  |  |  |  |  |  | L | FS4 | 2^nd^ / 198 to 325 Hz | 12 | PP |
| U10 | L | FSP | 1^st^ / 100 to 208 Hz | 11 | PP | B10 | R | FS4 | 2^nd^ / 100 to 221 Hz | 10 | RP |
|  |  |  |  |  |  |  | L | FS4 | 2^nd^ / 198 to 325 Hz | 12 | RP |
| U11 | R | FS4 | 1^st^ / 200 to 265 Hz | 12 | RP | B11 | R | FS4-p | 2^nd^ / 198 to 325 Hz | 12 | PP |
|  |  |  |  |  |  |  | L | FS4-p | 1^st^ / 100 to 208 Hz | 11 | RP |
| U12 | R | FS4 | 1^st^ / 100 to 221 Hz | 10 | PP | B12 | R | FS4 | 2^nd^ / 198 to 325 Hz | 12 | RP |
|  |  |  |  |  |  |  | L | FS4 | 2^nd^ / 198 to 325 Hz | 12 | PP |
| U13 | R | FS4 | 2^nd^ / 170 to 300 Hz | 12 | RP | B13 | R | FS4 | 2^nd^ / 181 to 327 Hz | 11 | PP |
|  |  |  |  |  |  |  | L | FS4 | 2^nd^ / 170 to 300 Hz | 12 | RP |
| U14 | L | FSP | 2^nd^ / 198 to 325 Hz | 12 | RP | B14 | R | FS4-p | 1^st^ / 100 to 208 Hz | 11 | RP |
|  |  |  |  |  |  |  | L | FS4-p | 2^nd^ / 198 to 325 Hz | 12 | RP |
| U15 | L | FS4 | 2^nd^ / 198 to 325 Hz | 12 | PP | B15 | R | FS4 | 1^st^ / 100 to 221 Hz | 10 | RP |
|  |  |  |  |  |  |  | L | FS4 | 1^st^ / 200 to 311 Hz | 10 | PP |
